# The Seroprevalence of Crimean-Congo Hemorrhagic Fever in Wild and Domestic Animals: An Epidemiological Update for Domestic Animals and First Seroevidence in Wild Animals from Turkiye

**DOI:** 10.3390/vetsci9090462

**Published:** 2022-08-29

**Authors:** Canakoglu Nurettin, Berber Engin, Tonbak Sukru, Aktas Munir, Vatansever Zati, Ozdarendeli Aykut

**Affiliations:** 1 Department of Virology, Milas Faculty of Veterinary Science, Muğla Sıtkı Koçman University, Muğla 48200, Türkiye; 2 Department of Biomedical and Diagnostic Sciences, College of Veterinary Medicine, University of Tennessee, Knoxville, TN 37996, USA; 3 Department of Virology, Faculty of Veterinary Medicine, Firat University, Elazığ 23119, Türkiye; 4 Department of Parasitology, Faculty of Veterinary Medicine, Firat University, Elazığ 23119, Türkiye; 5 Department of Parasitology, Faculty of Veterinary Medicine, Kafkas University, Kars 36100, Türkiye; 6 Vaccine Research, Development, and Application Center, Erciyes University, Kayseri 38280, Türkiye; 7 Department of Microbiology, Medical Faculty, Erciyes University, Kayseri 38280, Türkiye

**Keywords:** CCHFV, Crimean-Congo hemorrhagic fever, domestic animals, ELISA, hare, seroprevalence, wild animals, wild boar

## Abstract

**Simple Summary:**

Crimean-Congo hemorrhagic fever is a tick-borne and zoonotic emerging viral disease that is characterized by the sudden development of high fever and vascular bleeding in humans. The seroevidence among livestock has been reported, but it is less known in wild animals. Due to the importance and emerging state of the disease, we conducted a serosurvey on both domestic and wild animals in different areas of Turkiye. Serological investigations conducted on cattle, goats, and sheep revealed 10.81%, 15.15%, and 19.23% seropositivity, respectively, in the collected serum samples. We also found seropositivity rates in hare (23.81%) and wild boars (2.5%) indicating the substantial role of wild animals in virus epidemiology in Turkiye. This study provides first seroevidence of Crimean Congo hemorrhagic fever in wild animals in Turkiye.

**Abstract:**

Crimean-Congo hemorrhagic fever virus (CCHFV) is a zoonotic, tick-borne pathogen that is endemic to some parts of Europe, Africa, and Asia. The disease causes fever and hemorrhagic manifestations in humans but not in animals. Domestic and wild animals are asymptomatic hosts of CCHFV and are critical in the transmission cycle. *Hyalomma marginatum* spp. has been identified as the natural reservoir and vector of the virus in Turkiye. A few studies have been conducted on domesticated animals showing the seroprevalence of CCHFV in them, but seroevidence in wild animals is absent. For contributing this antrum to the understanding of virus transmission in Turkiye, we performed a seroprevalence investigation of CCHFV in both wild and domesticated animals in various geographical areas of Turkiye. In-house IgG iELISA was performed for the screening of sera IgG in a total of 582 animal samples collected from boar (n = 40), cattle (n = 259), goat (n = 132), hare (n = 21), and sheep (n = 130). Results from ELISA performed on domestic animals revealed 10.81%, 15.15%, and 19.23% anti-CCHF virus seropositivity in cattle, goats, and sheep, respectively, in collected serum samples. ELISA tests performed in wild animals showed 23.81% and 2.5% positivity in hare and wild boars, respectively, suggesting the importance of wild animals in CCHF virus epidemiology in Turkiye. This study performed the first serological investigation of CCHFV in wild animals and provided the first seroevidence of CCHFV in wild boars and hare in Turkiye.

## 1. Introduction

Crimean Congo hemorrhagic fever virus (CCHFV) is a tick-borne viral zoonotic virus that exclusively causes a disease in humans called the Crimean Congo hemorrhagic fever (CCHF) [1,2]. The disease is widely distributed in and across Asia, Africa, and some parts of Europe [3,4,5]. The virus is commonly transmitted by the vector and reservoir ticks under the *Hyalomma* genus [2]. In addition to infections from tick bites, humans can acquire the virus through direct contact with infected bodily fluids [3]. Farmers, slaughterhouse workers, and veterinarians working in endemic regions are primarily at risk of CCHF [6]. Nosocomial infections were also reported in intensive care units from people receiving therapy [7]. The disease typically starts with flu-like symptoms, with high fever and headache commonly reported after reports of tick bite. Some can develop petechial hemorrhagic symptoms, and soon after, bleeding from the body and hemorrhage become evident if the virus is not diagnosed and people are not treated early [1]. The mortality rate can vary between 5 and 40%. The options of therapy are limited to supportive therapy, and infected survivors often develop long-lasting immunity [1]. 

Currently, there is no specific antiviral therapy, but high-dose ribavirin administration has been found beneficial [8]. There is no approved vaccine against CCHFV, but a mice brain-derived and inactivated vaccine was developed by Bulgarian and Soviet scientists for military purposes [9]. However, the production of the virus from mice brains increases some autoimmune-like disease safety concerns, and the vaccine is not widely used. Recently, our preclinical studies using Vero cell-derived virus production and formalin inactivation have been successful, and a phase-I clinical study has been conducted without reporting any side effects [10,11].

The causative agent is characterized and identified as CCHFV [12]. The CCHF virus is a member of the largest RNA virus family, *Nairoviridae*, and is classified under the Orthonairovirus genus. The genome consists of three (S-small; M-Medium; L-Large) single-stranded (-) RNAs in a single virion. Viruses are encapsulated with nucleoprotein (NP)-coated RNAs and covered with lipid envelope that containing Gn and Gc spike glycoproteins [13]. 

The earliest CCHFV case was reported in the Crimean region with hemorrhagic manifestations in 1944. The disease or similar symptoms were steadily reported in humans from several regions of Southeast Europe, Asia, and Africa. In 1969, hemorrhagic fever manifestations causing virus showed morphological and serological similarities with that identified in Congo (1956), and hence, the virus and disease were known by the current name [12,14]. The first seroepidemiological IgG data from humans were reported in Turkiye (formerly known as Turkey) by Serter, but the first human cases were noticed later in Turkiye in 2002 [15,16]. Due to limited knowledge and lack of control measurements, the virus spread enormously in Turkiye, and soon after, the Kelkit Valley became an endemic region for CCHFV [3,16]. Several epidemiological investigations revealed that the viruses circulate in the endemic areas of Turkiye in an enzootic manner through the tick–vertebrate–tick cycle and that only humans show unprecedented infection [3]. CCHFV infection affected 10,562 cases between 2002 and 2017 with 4.74% mortality in Turkiye [17]. It was speculated that the virus circulates among wild and domesticated animals and is introduced to humans by tick bite [18]. *Hyalomma* spp. tick, particularly *Hyalomma marginatum*, and many others (*H. rufipes, H. anatolicum,* and *H. asiaticum*) have been identified as natural reservoirs and vectors of the virus; the distribution of disease occurrence in geographical areas closely resembles vector tick distribution [14,19]. In addition to the enzootic cycle of virus transmission, virus-infected vector ticks can transfer the virus by both transovarial and transstadial modes; therefore, the virus can be present in each life cycle of vector ticks and can pass to their offspring [20].

CCHFV has been identified in livestock animals including some wild mammals [18]. Although the virus causes transient viremia that lasts up to 15 days (*Lepus europaeus* by Zgurskaya et al.), sera IgG can be detectable longer [18,21]. Due to the lack of epidemiological results, regarding the seroprevalence of CCHFV in domestic and wild animals, our understanding of virus transmission in Turkiye is limited. Some results have been obtained from domesticated animals in Turkiye showing the seroprevalence of CCHFV to be between 13% and 66% from various domestic animals but with no seroprevalence in wild animals [22]. In this study, we performed a seroprevalence investigation of CCHFV in both wild (hares and boars) and domesticated animals (cattle, sheep, and goats) in various geographical areas of Turkiye. Epidemiological results from this study show that IgG seroprevalence of CCHFV in domestic and wild animals is 14.01% and 9.84%, respectively, in Turkiye. 

## 2. Material and Methods

### 2.1. Sample Collection and Ethics Statement

Ethical approval for this study was obtained from the local ethics committee (HDEE/FU; protocol no 40/07) and approved by the Institutional Board Committee of Firat University (CAR/FU protocol IP-1–13) and the Turkish Environmental Agency (TEA/Protocol 5199–3).The study was carried out in ten locations (Amasya, Bingol, Cankiri, Corum, Edirne, Erzurum, Kirklareli, Tekırdag, Tokat, Yozgat) of Turkiye commonly populated by *H. marginatum* ticks [23]. Samplings were performed between July 2008 and June 2011. Blood samples were directly collected from the jugular veins of domestic animals (cattle, sheep, goat) and obtained from hunted wild animals (hare, boar) by cardiac puncture. Blood collection was carried out in vacutainer tubes without an anticlotting agent. Serum samples were heat-inactivated at 56 °C for 30 min before performing IgG indirect IgG ELISA (iELISA). IgG iELISA was performed to screen a total of 582 animal samples collected from boar (n = 40), cattle (n = 259), goat (n = 132), hare (n = 21) and sheep (n = 130).

### 2.2. Virus, Cell Culture, and Antigen Production

The passage number 3rd Turkey-Kelkit06 endemic virus strain was propagated in susceptible Vero cells. Vero cells were maintained in 10% FBS containing DMEM F-12 media (1%Pen/Str/Amp added). Virus titer was performed as described previously, using pseudo-plaque assay [24]. Cells were infected with 0.01 MOI containing DMEM-F12 and no sera were used during the 60 min incubation time. Virus inoculum was washed away and replaced with 2% FBS containing DMEM F-12 medium. Flasks were kept in humidified 37°C incubators for 5 days and stored at −80 °C. The supernatant was pooled followed by three cycles of freeze–thaw, and pellets were discarded by centrifuge at 3.000 rpm for 30 min. Collected supernatants were subjected to PEG-NaCl precipitation. PEG8000 (50%) was used, and 15% and 10% of 23% NaCl was added to the final volume. Proteins were precipitated at 4 °C overnight on a slowly rotating magnetic stirrer. The virus was concentrated through a series of centrifugation steps that are described in an earlier study, and the resulting concentrated protein pellets were diluted with a Tris-EDTA-NaCl (TEN) buffer [10]. Virus inactivation was performed at room temperature for up to 7 days by the addition of 0.005% formaldehyde and used safely for coating ELISA plates. 

### 2.3. Indirect IgG ELISA (iELISA)

For the indirect IgG-ELISA, we have performed iELISA described before with minor modification [10,25]. CCHFV antigen produced and inactivated in Vero cells was bound to 96-well ELISA plates at 1 µg per well using pH 9.6 carbonate–bicarbonate buffer solution. After overnight incubation, the plate was triple washed with wash buffer (PBS solution containing 0.05% T20) and blocked with 5% skimmed milk in PBST. After one hour of incubation at 37 °C, the plates were washed again, as described in the previous step. Heat-inactivated (56 °C for 30 min) test sera were diluted for 1/100 in 5% skimmed milk in PBST for each animal species. Diluted sera were added to each well and incubated at 37 °C for 60 min. 

The plate was washed with wash buffer five times and then incubated with 1:5000 dilution of respective HRP-conjugated antibody for each animal species (Rabbit-anti-goat IgG: 6160-05; Goat-anti bovine IgG: 6030-05; Goat-anti-porcine IgG: 6050-05; Rabbit anti-sheep IgG: 6150-05; Goat anti-rabbit IgG:4030-05 acquired from Southern Biotech, Birmingham, AL, USA) in 0.05% PBST at 37 °C for 60 min. This was followed by washing five times with wash buffer, and the reaction was developed by adding 100 μL of TMB (Sigma-Aldrich, Saint Louis, MO, USA). Following incubation at room temperature for 10 min, the reaction was stopped using 100 μL of 1N HCl as a stop solution, and the absorbance was recorded at 450 nm using an ELISA plate reader (Thermo, Waltham, MA, USA). The cut-off values were determined using negative serum panels that were confirmed using Vector-best (Novosibirsk, Russia) IgG ELISA and Western blotting (data not shown) from each animal species (n = 3). The cut-off value was calculated as the multiplication of geometric mean (GMT) OD value by 2.5 [26,27].

### 2.4. Statistical Analysis

GraphPad Prism7 (Carlsbad, CA, USA) was used to compare the significance levels between the wild and domestic animals. A chi-square with Fisher’s exact test was performed for statistical analysis. For the percent coefficient of variability (CV%) of ELISA, the standard deviation (SD) of mean OD of the replicates was calculated and the results expressed as percentage to SD over the mean [CV = (SD/Mean) × 100].

## 3. Results

All virus production and live virus handling took place in BSL-3 laboratories located at Firat University, Virology department (Elazig, Turkiye). An in-house iELISA protocol was followed as described and optimized for IgG detection from different animal species [10,25]. The cut-off values were determined using true negative serum samples for each animal species. The samples were previously determined negative, confirmed by Western blotting followed by commercial IgG ELISA (data not shown), and cut-off values were calculated as follows: boar ≥ 0.13, cattle ≥ 0.15, sheep ≥ 0.11, hare ≥ 0.11. The validity and specificity of in-house iELISA were shown before [10]. For the accuracy and consistency of IgG results, we calculated CV% of ELISA for boar (7.60%), cattle (9.84%), goat (9.69%), hare (5.27%), and sheep (6.19%). Results showed that the variances between two replicates were lower than 10% in all animal serum samples being tested [28].

**Figure 1:**
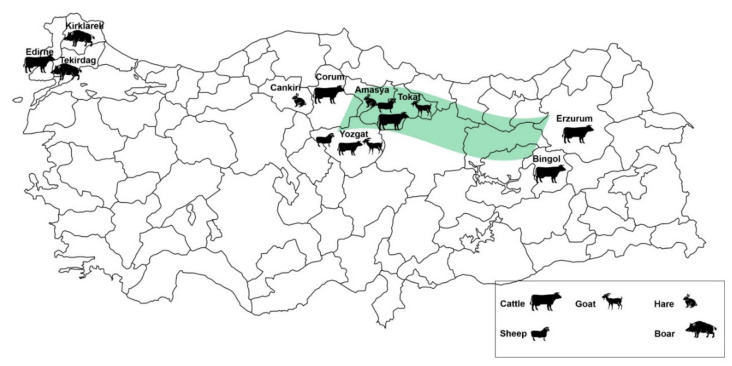
The study area of wild and domestic animals in Turkiye. The green represents the endemic region (Kelkit Valley) of CHHFV in Turkiye.

**Figure 2:**
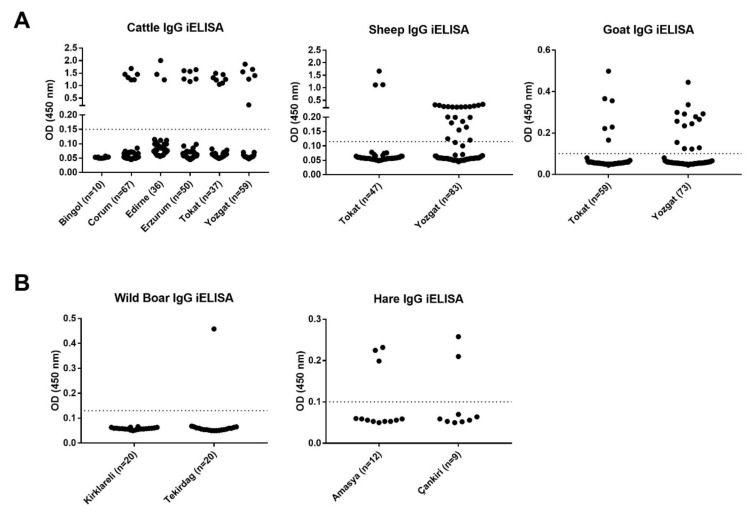
Indirect IgG results showing OD results obtained from iELISA. An optimized in-house iELISA was performed on heat (56 °C) inactivated serum samples. Serum samples were collected from domestic (**A**) and wild animals (**B**) located in different regions of Turkiye (cattle iELISA cut-off: 0.15, sheep iELISA cut-off: 0.11, goat iELISA cut-off: 0.11, wild boar iELISA cut-off: 0.13, hare ELISA cut-off: 0.11). Dotted line represents the OD cut-off threshold (GMTx2.5 of negative assay controls) and black dots represent individual OD values.

Serum samples were obtained from various geographic areas, as presented in Figure 1. According to the iELISA results (Figure 2), domesticated animals showed 14.01% IgG seropositivity in total (Table 1). Among the screened serum samples in domestic animals, higher results were taken from sheep (19.23%) followed by goat (15.15%), while cattle showed 10.81%. CCHFV circulates in wild fauna through vector ticks, and ticks play an important role between domestic and wild mammals in terms of virus transmission. IgG presence was detected in a total of 9.84% of wild animals (Table 2). Sera collected from wild boars showed 2.5% seropositivity, which was 23.81% in hare. There was no significant difference in IgG seropositivity compared between wild and domestic animals (*p* = 0.367). However, seropositivity collected from sheep showed a significant difference (** *p* < 0.01) between Tokat and Yozgat cities, and this significance was higher in Yozgat (26.51%) than in Tokat (6.38%). 

## 4. Discussion

Seroepidemiological studies of CCHFV in animals provide evidence of the virus circulating in endemic regions and also help to identify the risk areas. Anti-CCHFV IgG has been detected in both domestic and wild animals in various endemic regions. Seroprevalence results without symptomatic infection from various animals show that animals can be infected with CCHFV. Serum IgG positivity in animals can last longer than asymptomatic viremia (7–15 days) [18,21]. Serological evidence was commonly reported from domestic animals including cattle, sheep, goats, camels, and buffalo. Some investigations also evidenced anti-CCHFV IgG seroprevalence in wild animals including hares, hedgehogs, warthogs, foxes, zebras, and African buffaloes. Except for some species (guinea fowls and ostriches), seropositivity has not been identified in birds [18]. In this study, we provided an epidemiological analysis of CCHFV seropositivity among several domestic and wild animal species from Turkiye. Serum samples were collected from various wild (hare, boar) and domestic animals (cattle, sheep, and goat) to identify the IgG seropositivity.

To measure seropositivity, a reverse passive hemagglutination-inhibition assay, virus neutralization assay, agar-gel diffusion precipitation, indirect immunofluorescence assay, and IgG ELISA were carried out for seroprevalence of CCHFV in animals [18]. Due to several concerns such as the safety (does not require working with live virus), reliability, and consistency of assays, IgG ELISA has several advantages over the other assays. CCHFV NP is highly conserved among the serogroups and even in the *Nairoviridae* family. In fact, viral NP is the most abundant protein and is also known as an immune-dominant protein [29]. This unique antigen identity led researchers to produce recombinant NP-based iELISA [30]. Marriott et al. showed that of all seven serogroups that were tested against recombinantly expressed viral CCHFV NP in ELISA, only those antibodies raised against CCHFV virus strains reacted in CCHFV ELISA and a weak Hazara virus IgG response were observed [29]. In a recent study, it was observed by Kalkan-Yazici et al. that even though CCHFV NP is known as the most conserved viral protein in *Nairoviridae*, there is a cross-reaction between Hazara virus NP and CHFV NP due to antigenic similarities [31]. However, neither cattle nor sheep were found to be susceptible to Hazara virus infection, indicating that the cross-reactivity of anti-CCHFV NP IgG with other serogroups is limited [32]. Several other studies that produced CCHFV NP using recombinant systems also indicated that NP-based ELISA have several advantages over the other assays even more sensitive than immunofluorescence assays to distinguish other serogroups including Nairobi sheep disease virus [33,34]. Nevertheless, Gulce-Iz et al. showed high sensitivity of recombinant NP and viral mucin-like protein coated ELISA using convalescent human serum samples, but its sensitivity lowered from 97% to 73% when acute phase serum samples being tested showing the need to use more than one viral antigen for IgG capture and ELISA plate coating [30]. Therefore, in this study, we coated ELISA plates with intact whole virus but with formalin-inactivated antigens to detect serum IgGs (sensitivity and specificity measured as 94% and 95%, respectively, using mice sera-data not shown). However, we did not test cross-reactivity with other serogroups, showing the limitations of our results. In spite of that, in our previous studies we observed that the majority of viral proteins produced from Vero cells, which were similarly produced in this study, were viral NPs [10]. Here, we modified our iELISA to be animal specific by replacing the conjugate, and we set cut-off values based on negative serum samples that were confirmed in Western blotting (data not shown). 

The serological investigations were carried out on domestic animals from different countries, and the results were summarized by Spengler et al. [18]. The number of studies among the wide domestic animal species around the world indicates that cattle are most commonly investigated for anti-CCHFV antibodies, followed by sheep and goats. The estimated average seroprevalence of CCHFV in cattle, sheep, and goats is 19.33%, 23.85%, and 28.07%, respectively, and these seropositivity rates are higher than our seropositivity results found in cattle (10.81%), sheep (19.23%), and goats (15.15%). A recent study conducted in the Central Anatolia district of Turkiye detected seropositivity in cattle at 1.2% of 329 serum samples collected using commercial human IgG ELISA with some modifications [35]. 

Another seroprevalence study conducted in the Marmara district of Turkiye estimated 13%, 31.8%, and 66% seropositivity in serum samples collected from cattle, sheep, and goats, respectively, using serum neutralization assay [22]. Sheep and goats are the most widely produced livestock in Turkiye followed by cattle. Albayrak et al. conducted a serosurvey in sheep and goats by screening serum samples with a modified commercial ELISA. A study carried out in the Northern District of Turkiye reported seropositivity rates of 85.71% and 66.66% in sheep and goats, respectively [36]. These higher seropositivity disturbances could be influenced by geographical locations, which determine the climate changes and in turn the vector tick distributions. In some parts of our serosurvey area, the study was conducted on domestic animals (Corum, Tokat, and Yozgat cities) located within or neighboring the Kelkit Valley, which is the endemic region for CCHFV in Turkiye. A recent study carried out in neighboring endemic foci of Kelkit Valley in Turkiye showed 36.21%, 6.27%, and 6.67% seropositivity rates in cattle, sheep, and goats, respectively, using a modified commercial IgG ELISA [37]. Even though we determined 6.38% seropositivity in sheep located in Tokat city, the IgG seropositivity estimated significantly lower (** *p* < 0.01) when compared with Yozgat (26.51%). Such differences can be explained by ages of the animal sera collected, the foci of vector ticks, and the wild small animal population (hare), and even many other factors should be taken in consideration. For example, Tonbak et al. studied the distribution of tick species located in endemic foci-Kelkit Valley of CCHFV and identified that the most abundant tick species in domestic animals (cattle, sheep, and goats) was Rhipicephalus bursa followed by *Hyalomma marginatum marginatum* [38]. Such disturbances can also affect the seropositivity found in animals. The reasons for such variations in seropositivity in domestic animals are unclear; however, many factors including the time of sample collection (after the occurrence of infection); the ages of animals; the management and care of livestock; and the locations of the serum sampling, assays, and screening can influence the study results of seroprevalence. In addition, the lack of standardized tests should be considered a potential explanation for variations in seropositivity.

Although Turkiye has several wild animals in nature, no study has conducted a serological investigation of CCHFV in wild animals. In this study, we also evaluated the seroepidemiology of CCHFV in animals by collecting serum samples from hare and wild boars. If we consider smaller animals such as hares to serve as a host for immature ticks, it is not surprising that hares were found seropositive (23.81%) in this study. Early investigations by Zgurskaya et al. evidenced that *Hyalomma m. marginatum* tick species fed on infected hares (*Lepus europaeus*) and the virus was transmitted through transovarial and transstadial cycle in an experimental infection [39]. In the same study, Zgurskaya et al. also demonstrated that viremia (7–15 days) exists and that antibodies developed in hares after CCHFV infection [39]. Serological studies in hares conducted in countries neighboring Turkiye such as Bulgaria (3%) and Iran reported serological prevalence in wild hares (*Lapus* spp.) [18]. Seropositivity in wild rabbits (European hare) was also reported in Russia (20%) and Hungary (6%) [2,40]. Additionally, several studies in different countries in Asia and Africa evidenced seroprevalence in wild hares, which varied from 3% to 40% [2,18]. On the contrary, a study could not find a seropositive in samples collected from European rabbits in Spain, where human cases were reported since 2013 [41]. These findings reported in Spain remain unclear in terms of the role of the hare in CCHFV epidemiology. We could support the previous evidence by suggesting the role of the hare in CCHFV transmission might not be direct and that they potentially serve as amplifier hosts contributing to maintain the virus in wildlife. Curiously, Hoogstraal stated that low density in the population of hares and some other rodents, caused by flooded burrows (1945) in the Crimean region, might have “normalized” the *H. m. marginatum* tick species in the field, and hence, human attack cases dropped from 100 to around 9 in the subsequent year. It was highlighted in the same review that adult ticks will most likely attack humans where inadequate large animals are prevalent [2]. Some others also claimed that both wild boars and hare densities have increased noticeably in the areas of Turkiye, where most of the CCHF cases have occurred [42]. Some studies claimed that CCHFV human cases are silent when small and large animal populations are adequate for vector ticks, but outbreaks or sporadic human cases occur when large animals are not adequate for unfed-questing ticks. This theory can explain why adult ticks prefer humans in the absence of large animals, but comprehensive epidemiological investigations are needed to support the idea [18,43].

Wild boars are considered large animals in the enzootic cycle of adult ticks. However, the number of serological studies is not satisfactory. This scarcity leaves a gap in knowledge regarding the role of wild boars in CCHFV epidemiology. Serological investigations from South Africa determined a 5% seropositivity ratio in warthogs [44]. Nevertheless, a different study showed no seropositivity in warthogs in South Africa [45]. In our study, we have found 2.5% seropositivity in wild boars out of the 40 samples collected in the Thrace district of Turkiye. In a recent study, sera samples collected from wild boar in Catalonia and Ebro Delta of Spain showed 3.20% and 20.83% IgG positivity, respectively. In the same study, other than wild boar samples, Spanish wild goats (*Iberian ibex*) showed higher seropositivity rates of 78.57% and 100% in Spain [41]. The reasons for such huge differences between two wild animals (wild boars vs Spanish wild goats) are not clear but suggest a possible role of wild animals as much as livestock in introducing CCHFV in non-endemic zones.

To understand CCHFV epidemiology, wild animals and domestic animals need to be surveyed, since vector and reservoir ticks circulate the tick–host–tick lifecycle. Immature ticks feed preferentially on small animals (hares, hedgehogs, birds), while adult ticks prefer larger mammals (cattle, sheep, goats, pigs, and wild boar) [2]. Based on this fact, serological investigations of various animal species should be considered in the endemic areas where small and large animals co-exist. In our study, we could not find a significant difference between wild animals and domestic animals in terms of seropositivity (9.84% and 14.01%, respectively) in Turkiye, suggesting that both domestic and wild animals have an essential role in CCHFV transmission. It is important to note that the very small sample size of wildlife in this study makes the percent of seropositives appear higher than it may actually be. Further studies should focus on providing more serological data by screening more wild animals from different CCHFV foci in Turkiye as well as from different countries.

## 5. Conclusions

In this study, we provided the first seroprevalence results of CCHFV in wild animals, and we evidenced the serum IgG positivity in hares (23.81%) and wild boars (2.5%), which was comparable with domestic animals. We also screened domestic animals for IgG prevalence, and compared our results with those for wild animals (14.01% vs 9.84%, respectively) indicating that wild animals and livestock are equally important for circulating the CCHF virus in endemic areas such as seen in Turkiye.

## Figures and Tables

**Table 1:** Numbers and IgG iELISA seropositivity of CCHFV in domestic animals collected from various locations of Turkiye.

		Animal	Location	Date of Sampling	IgG Seropositivity (%)	Positive/Total (n/n)	*p*-Value
		Cattle	Bingol	June 2010	0.00	0/10	0.506
			Corum	July 2009	8.96	6/67	
			Edirne	April 2010	8.33	3/36	
			Erzurum	August 2009	12.00	6/50	
			Tokat	July 2009	18.92	7/37	
			Yozgat	July 2009	10.17	6/59	
	Sub-total				10.81	28/259	
		Goat	Tokat	July 2009	10.17	6/59	0.151
			Yozgat	August 2009	19.18	14/73	
	Sub-total				15.15	20/132	
		Sheep	Tokat	July 2008	6.38	3/47	0.005
			Yozgat	July 2008	26.51	22/83	
	Sub-total				19.23	25/130	
Total					14.01	73/521	

**Table 2:** Numbers and IgG iELISA seropositivity of CCHFV in wild animals collected from various locations of Turkiye.

		Animal	Location	Date of Sampling	IgG Seropositivity (%)	Positive/Total (n/n)	*p*-Value
		Boar	Kirklareli	March 2010	0.00	0/20	0.311
			Tekirdag	March 2010	5.00	1/20	
	Sub-total				2.50	1/40	
		Hare	Amasya	June 2011	25.00	3/12	0.882
			Cankiri	June 2011	22.22	2/9	
	Sub-total				23.81	5/21	
Total					9.84	6/61	

## Data Availability

Not applicable.

## References

[1] Ergönül Ö. (2006). Crimean-Congo haemorrhagic fever. Lancet Infect. Dis..

[2] Hoogstraal H. (1979). The Epidemiology of Tick-Borne Crimean-Congo Hemorrhagic Fever in Asia, Europe, and Africa. J. Med. Entomol..

[3] Ergonul O. (2012). Crimean–Congo hemorrhagic fever virus: New outbreaks, new discoveries. Curr. Opin. Virol..

[4] Bente D.A., Forrester N.L., Watts D.M., McAuley A.J., Whitehouse C.A., Bray M. (2013). Crimean-Congo hemorrhagic fever: History, epidemiology, pathogenesis, clinical syndrome and genetic diversity. Antivir. Res..

[5] Shahhosseini N., Wong G., Babuadze G., Camp J.V., Ergonul O., Kobinger G.P., Chinikar S., Nowotny N. (2021). Crimean-Congo Hemorrhagic Fever Virus in Asia, Africa and Europe. Microorganisms.

[6] Mourya D.T., Yadav P.D., Gurav Y.K., Pardeshi P.G., Shete A.M., Jain R., Raval D.D., Upadhyay K.J., Patil D.Y. (2019). Crimean Congo hemorrhagic fever serosurvey in humans for identifying high-risk populations and high-risk areas in the endemic state of Gujarat, India. BMC Infect. Dis..

[7] Tsergouli K., Karampatakis T., Haidich A.B., Metallidis S., Papa A. (2020). Nosocomial infections caused by Crimean–Congo haemorrhagic fever virus. J. Hosp. Infect..

[8] Ozbey S.B., Kader Ç., Erbay A., Ergönül Ö. (2014). Early Use of Ribavirin Is Beneficial in Crimean-Congo Hemorrhagic Fever. Vector Borne Zoonotic Dis..

[9] Papa A., Papadimitriou E., Christova I. (2011). The Bulgarian vaccine Crimean-Congo haemorrhagic fever virus strain. Scand. J. Infect. Dis..

[10] Berber E., Çanakoğlu N., Tonbak Ş., Ozdarendeli A. (2021). Development of a protective inactivated vaccine against Crimean–Congo hemorrhagic fever infection. Heliyon.

[11] Erenmemisoglu A. Phase I Study to Evaluate Basic Pharmacodynamic, Pharmacological and Toxicological Effects of the Newly Developed Crimean-Congo Hemorrhagic Fever Vaccine for Humans. https://clinicaltrials.gov/ct2/show/NCT03020771.

[12] Chumakov M.P., Smirnova S.E., Tkachenko E.A. (1970). Relationship between strains of Crimean haemorrhagic fever and Congo viruses. Acta Virol..

[13] Adams M.J., Lefkowitz E.J., King A.M.Q., Harrach B., Harrison R.L., Knowles N.J., Kropinski A.M., Krupovic M., Kuhn J.H., Mushegian A.R. (2017). Changes to taxonomy and the International Code of Virus Classification and Nomenclature ratified by the International Committee on Taxonomy of Viruses (2017). Arch. Virol..

[14] Grobov A.G. (1946). Carriers of Crimean Haemorrhagic Fever. Med. Parazitol..

[15] Serter D. (1980). Present status of arbovirus sero-epidemiology in the Aegean region of Turkey. Zentralbl. Bakteriol..

[16] Karti S.S., Odabasi Z., Korten V., Yilmaz M., Sonmez M., Caylan R., Akdogan E., Eren N., Koksal I., Ovali E. (2004). Crimean-Congo hemorrhagic fever in Turkey. Emerg. Infect. Dis..

[17] Halk Sagligi Genel Mudurlugu, Turkiye Mortality and Morbidity of CCHFV in Turkey between 2008 and 2017. https://hsgm.saglik.gov.tr/tr/zoonotikvektorel-kkka/zoonotikvektorel-kkka-istatistik.

[18] Spengler J.R., Bergeron É., Rollin P.E. (2016). Seroepidemiological Studies of Crimean-Congo Hemorrhagic Fever Virus in Domestic and Wild Animals. PLoS Negl. Trop. Dis..

[19] Korshunova O., Plontkovskaya S. (1949). About the Virus isolated from the Ticks Hyalomma marginatum marginatum Koch. Zool. Zhurnal.

[20] Shepherd A.J., Swanepoel R., Shepherd S.P., Leman P.A., Mathee O. (2009). Viraemic transmission of Crimean-Congo haemorrhagic fever virus to ticks. Epidemiol. Infect..

[21] Spengler J.R., Estrada-Peña A., Garrison A.R., Schmaljohn C., Spiropoulou C.F., Bergeron É., Bente D.A. (2016). A chronological review of experimental infection studies of the role of wild animals and livestock in the maintenance and transmission of Crimean-Congo hemorrhagic fever virus. Antivir. Res..

[22] Tuncer P., Yesilbag K., Alpay G., Dincer E., Girisgin A.O., Aydin L., Yavuz U., Ozkul A. (2014). Crimean-Congo hemorrhagic fever infection in domestic animals in Marmara region, Western Turkey. Ank. Univ. Vet. Fak. Derg..

[23] Hekimoglu O., Ozer A.N. (2017). Distribution and phylogeny of Hyalomma ticks (Acari: Ixodidae) in Turkey. Exp. Appl. Acarol..

[24] Berber E., Canakoglu N., Yoruk M.D., Tonbak S., Aktas M., Ertek M., Bolat Y., Kalkan A., Ozdarendeli A. (2013). Application of the pseudo-plaque assay for detection and titration of Crimean-Congo hemorrhagic fever virus. J. Virol. Methods.

[25] Canakoglu N., Berber E., Tonbak S., Ertek M., Sozdutmaz I., Aktas M., Kalkan A., Ozdarendeli A. (2015). Immunization of Knock-Out α/β Interferon Receptor Mice against High Lethal Dose of Crimean-Congo Hemorrhagic Fever Virus with a Cell Culture Based Vaccine. PLoS Negl. Trop. Dis..

[26] Garcia S., Chinikar S., Coudrier D., Billecocq A., Hooshmand B., Crance J.M., Garin D., Bouloy M. (2006). Evaluation of a Crimean-Congo hemorrhagic fever virus recombinant antigen expressed by Semliki Forest suicide virus for IgM and IgG antibody detection in human and animal sera collected in Iran. J. Clin. Virol..

[27] Rangunwala A., Samudzi R.R., Burt F.J. (2013). Detection of IgG antibody against Crimean-Congo haemorrhagic fever virus using ELISA with recombinant nucleoprotein antigens from genetically diverse strains. Epidemiol. Infect..

[28] Reed G.F., Lynn F., Meade B.D. (2002). Use of coefficient of variation in assessing variability of quantitative assays. Clin. Diagn. Lab. Immunol..

[29] Marriott A.C., Polyzoni T., Antoniadis A., Nuttall P.A. (1994). Detection of human antibodies to Crimean-Congo haemorrhagic fever virus using expressed viral nucleocapsid protein. J. Gen. Virol..

[30] Gülce-İz S., Elaldı N., Can H., Şahar E.A., Karakavuk M., Gül A., Kumoğlu G.Ö., Döşkaya A.D., Gürüz A.Y., Özdarendeli A. (2021). Development of a novel recombinant ELISA for the detection of Crimean-Congo hemorrhagic fever virus IgG antibodies. Sci. Rep..

[31] Kalkan-Yazıcı M., Karaaslan E., Çetin N.S., Hasanoğlu S., Güney F., Zeybek Ü., Doymaz M.Z. (2021). Cross-Reactive Anti-Nucleocapsid Protein Immunity against Crimean-Congo Hemorrhagic Fever Virus and Hazara Virus in Multiple Species. J. Virol..

[32] Hartlaub J., von Arnim F., Fast C., Somova M., Mirazimi A., Groschup M.H., Keller M. (2020). Sheep and Cattle Are Not Susceptible to Experimental Inoculation with Hazara Orthonairovirus, a Tick-Borne Arbovirus Closely Related to CCHFV. Microorganisms.

[33] Hartlaub J., Daodu O.B., Sadeghi B., Keller M., Olopade J., Oluwayelu D., Groschup M.H. (2021). Cross-Reaction or Co-Infection? Serological Discrimination of Antibodies Directed against Dugbe and Crimean-Congo Hemorrhagic Fever Orthonairovirus in Nigerian Cattle. Viruses.

[34] Lombe B.P., Miyamoto H., Saito T., Yoshida R., Manzoor R., Kajihara M., Shimojima M., Fukushi S., Morikawa S., Yoshikawa T. (2021). Purification of Crimean–Congo hemorrhagic fever virus nucleoprotein and its utility for serological diagnosis. Sci. Rep..

[35] Şevik M. (2018). Epidemiological investigation of Crimean-Congo hemorrhagic fever infection in cattle in some provinces of Turkey. J. Etlik Vet. Microbiol..

[36] Albayrak H., Ozan E., Kurt M. (2012). Serosurvey and molecular detection of Crimean–Congo hemorrhagic fever virus (CCHFV) in northern Turkey. Trop. Anim. Health Prod..

[37] Ozan E., Ozkul A. (2020). Investigation of Crimean-Congo hemorrhagic fever virus in ruminant species slaughtered in several endemic provinces in Turkey. Arch. Virol..

[38] Tonbak S., Aktas M., Altay K., Azkur A.K., Kalkan A., Bolat Y., Dumanli N., Ozdarendeli A. (2006). Crimean-Congo Hemorrhagic Fever Virus: Genetic Analysis and Tick Survey in Turkey. J. Clin. Microbiol..

[39] Zgurskaya G., Berezin V., Smirnova S., Chumakov M. (1971). Investigation of the question of Crimean hemorrhagic fever virus transmission and interepidemic survival in the tick Hyalomma plumbeum plumbeum Panzer. Tr. Inst. Polio Virusn. Entsefalitov Akad. Med. Nauk SSSR.

[40] Németh V., Oldal M., Egyed L., Gyuranecz M., Erdélyi K., Kvell K., Kalvatchev N., Zeller H., Bányai K., Jakab F. (2013). Serologic evidence of Crimean-Congo hemorrhagic fever virus infection in Hungary. Vector Borne Zoonotic Dis..

[41] Espunyes J., Cabezón O., Pailler-García L., Dias-Alves A., Lobato-Bailón L., Marco I., Ribas M.P., Encinosa-Guzmán P.E., Valldeperes M., Napp S. (2021). Hotspot of Crimean-congo hemorrhagic fever virus seropositivity in wildlife, northeastern Spain. Emerg. Infect. Dis..

[42] Vatansever Z., Uzun R., Estrada-Pena A., Ergonul O., Ergonul O., Whitehouse C.A. (2007). Crimean-Congo Hemorrhagic Fever: A Global Perspective.

[43] Tsokana C.N., Sokos C., Giannakopoulos A., Birtsas P., Valiakos G., Spyrou V., Athanasiou L.V., Rodi Burriel A., Billinis C. (2020). European Brown hare (Lepus europaeus) as a source of emerging and re-emerging pathogens of Public Health importance: A review. Vet. Med. Sci..

[44] Shepherd A.J., Swanepoel R., Shepherd S.P., McGillivray G.M., Searle L.A. (1987). Antibody to Crimean-Congo hemorrhagic fever virus in wild mammals from southern Africa. Am. J. Trop. Med. Hyg..

[45] Burt F.J., Swanepoel R., Braack L.E.O. (2009). Enzyme-linked immunosorbent assays for the detection of antibody to Crimean-Congo haemorrhagic fever virus in the sera of livestock and wild vertebrates. Epidemiol. Infect..

